# Understanding older adults’ adoption of smart product-service systems for medication management in China: an extended UTAUT model approach

**DOI:** 10.3389/fpubh.2025.1657473

**Published:** 2025-10-03

**Authors:** Ruochen Fu, Xiaofei Ma

**Affiliations:** ^1^Computing, Goldsmiths, University of London, London, United Kingdom; ^2^College of Art, Beijing Union University, Beijing, China

**Keywords:** smart product-service systems, medication management, older adults, extended UTAUT, technology acceptance, China

## Abstract

The rapid aging of China’s population is intensifying the burden of chronic disease management and driving a shift toward home-based care supported by Smart Product-Service Systems (SPSS), such as smart pillboxes and medication management applications. However, the success of these technologies largely depends on their acceptance and use by older adults. This study aims to explore the behavioral factors influencing the adoption of SPSS for medication management among older adult users in China, using an extended Unified Theory of Acceptance and Use of Technology model. To better capture the complex interactions between the service delivery environment and users’ personal capabilities and psychological states, the model incorporates three additional constructs: Self-Efficacy, Health Consciousness, and Medical Service Satisfaction. Structural Equation Modeling based on Partial Least Squares was employed to validate the model using empirical data collected from older adult respondents in China. The results show that Performance Expectancy, Effort Expectancy, Social Influence, Self-Efficacy, Health Consciousness, and Medical Service Satisfaction all significantly influence behavioral intention, whereas Facilitating Conditions did not demonstrate a significant effect. These findings highlight that, within the Chinese cultural context, family support and intrinsic health motivation are more critical than external infrastructure in shaping technology adoption. This study contributes to a deeper theoretical understanding of health technology adoption among older adults and offers practical insights for SPSS developers. It underscores the importance of culturally adapted and user-friendly smart systems to improve medication adherence and health outcomes in aging societies.

## 1 Introduction

The global trend of population aging is reshaping demographic structures worldwide, triggering profound socio-economic transformations. The growing societal pressures associated with aging are becoming increasingly evident, placing unprecedented demands on healthcare systems particularly in the area of chronic disease management (1). In response to the healthcare challenges posed by an aging population, the Chinese government has initiated a reallocation of public medical resources, emphasizing the development of home-based healthcare models to alleviate the burden on institutional care facilities. This shift has transferred the responsibility of medication management to older adult individuals themselves, leading to a significant rise in the need for self-managed medication at home.

The Smart Product Service System (SPSS), as an emerging technological solution, fundamentally differs from traditional single-technology products. SPSS integrates intelligent hardware (e.g., smart pill boxes) with continuous digital services (such as medication reminders, health monitoring, and remote consultations), creating a dual value system of “product + service.” (2). This integrative characteristic makes the adoption process of SPSS more complex than traditional technologies: users are required not only to learn how to use the hardware but also to continuously interact with the service system, establishing long-term usage habits. For older adult users, establishing this ongoing service relationship demands a higher level of technological learning ability and sustained willingness to engage (3).

However, the effectiveness of technological applications in practice largely depends on the acceptance and willingness of the target users to adopt them. Research has shown that even well-developed healthcare technologies may fail to achieve their intended outcomes if user acceptance is low (4). Among older adults, the process of technology adoption is particularly complex, often influenced by a combination of age-related cognitive changes, health conditions, and cultural background (5, 6) examined older adults’ perceptions of the usability of mobile medication management applications. Similarly, Minaam et al. (7) explored the feasibility of enhancing caregivers’ medication management strategies through technological interventions; however, comparatively less attention has been paid to the acceptance of such technologies by older adult users themselves.

Existing research on technology adoption among older adults primarily focuses on Western developed countries, but the technology adoption behaviors of older adult individuals in China may exhibit significantly different characteristics. First, there is a marked difference in cultural background. China’s collectivist culture emphasizes family support and intergenerational dependence, whereas Western societies are rooted in individualistic values, prioritizing independence and autonomy (8). Secondly, the digital literacy of older adults affects their use of digital health resources (9, 10). The digital literacy and e-health literacy of the older adults in China are generally low, hindering the development of digital care services for older adults (11). Lastly, the distribution of healthcare resources in China is uneven. Compared to the more developed and transparent public healthcare systems in Western countries, older adult individuals in China rely on institutional safeguards for trust in public healthcare services, while being deeply influenced by personal experiences and social networks (12, 13). These differences suggest that directly applying Western theoretical frameworks may not accurately explain the SPSS adoption behaviors of older adult individuals in China.

The Unified Theory of Acceptance and Use of Technology (UTAUT) has been widely adopted due to its broad applicability in predicting user behavior (9). In the healthcare domain, researchers have frequently applied the UTAUT model to examine the intention of medical professionals in developing countries to use e-health services (14). However, the majority of these studies have primarily focused on the technological utility of such systems or have adopted the perspectives of healthcare providers, rather than end-users. For example, Edo et al. (15) assessed the impact of digital health technologies on the healthcare industry and explored the factors influencing healthcare professionals’ adoption of digital technologies. Similarly, Diel et al. (16) investigated physicians’ acceptance of telemedicine for online consultations. Bunnell et al. (17) analyzed the acceptability of telemedicine through the characteristics of mental health professionals (18). These studies primarily focus on the adoption of emerging technologies by healthcare professionals, with limited attention given to the perspectives of older adult users. The existing research has not fully considered the service attributes of SPSS. The traditional UTAUT model primarily addresses one-time technology adoption decisions, whereas SPSS requires users to establish an ongoing service usage relationship, involving unique factors such as service quality perception, health status, and the establishment of sustained efficacy.

Based on this research gap, this study proposes the following specific research questions:

1. What factors influence the willingness of older adult users people to adopt intelligent medication management systems (e.g., smart pillboxes and associated service applications) in the context of Chinese culture?
2. How do these influencing mechanisms differ from findings in Western studies, and what implications do they have for theoretical development?

To address this research gap, the study extends the original UTAUT model by incorporating Health Consciousness (HC) to capture the intrinsic motivation for active health management, Self-Efficacy (SE) to assess confidence thresholds in technology use, and Medical Service Satisfaction (MSS) to explore how service quality and subjective experiences may impact technology adoption. This extension not only responds to the call by Martins et al. (19) to adapt technology adoption models to specific contextual needs, but also enriches the application of UTAUT in service system contexts by incorporating the service attributes of SPSS into the technology adoption framework. It reveals the unique pathways of technology adoption among the older adults in the context of Chinese culture, providing new theoretical perspectives for research on technology adoption by the older adults in developing countries. Additionally, it offers empirical evidence for the design optimization and promotional strategy formulation of intelligent medication management systems.

## 2 Extension of UTAUT

Venkatesh et al. (20) conducted an empirical investigation and a comprehensive review of relevant studies. By synthesizing the constructs from eight previously established models of behavioral intention in the context of technology acceptance, they developed the Unified Theory of Acceptance and Use of Technology (UTAUT) model. As illustrated, the UTAUT model comprises six core constructs. In addition, Venkatesh et al. (20) proposed four moderating variables gender, age, experience, and voluntariness of use to enhance the model’s predictive power. Using the same dataset, the UTAUT model outperformed the other eight models, accounting for nearly 70% of the variance in behavioral intention and 50% of the variance in technology use (21). Therefore, the UTAUT model serves as the theoretical foundation for this study’s investigation into older adults’ use of SPSS.

In the field of mobile health (mHealth), the UTAUT model has been widely applied to investigate technology acceptance behaviors across diverse populations. For example, Hoque and Sorwar (9) examined the willingness of older adults in Bangladesh to adopt mHealth services, while Shiferaw et al. (22) analyzed healthcare providers’ adoption of telemedicine technologies. Although UTAUT demonstrates strong predictive power, emerging evidence suggests that its original constructs may be insufficient when applied to SPSS, particularly among the older adults population (23, 24).

The SPSS differs fundamentally from traditional technology products. The value of traditional technology products is embedded in the product during the production phase, and users obtain value directly through product usage. In contrast, SPSS is characterized by the integration of tangible products and intangible services through digital platforms. This hybrid nature is service-dominant and requires continuous interaction between users and service providers to co-create value (25, 26). This difference is particularly evident in the field of chronic disease management. The treatment outcomes and health improvements for patients require sustained use over weeks or even months to gradually manifest (27, 28). Unlike the immediate functional value provided by traditional medical devices, the value realization of SPSS exhibits a significant delay. This extended timeline for value realization presents dual challenges: on one hand, it increases the uncertainty of user decisions, as users struggle to assess the system’s actual effects during the initial adoption phase; on the other hand, it requires users to possess stronger self-management skills and sustained motivation to maintain long-term system use and behavioral changes in health (29). For older adults patients with chronic diseases, this challenge is even more pronounced. The value co-creation process requires older adult users to actively participate in data sharing, health monitoring, and behavior modification, which fundamentally differs from the passive healthcare service model they are accustomed to Tang et al. (30). In addition, the older adults population tends to rely more on the continuous support and guidance of service providers, making the quality of the service relationship a key factor influencing their adoption and sustained usage (31). Meanwhile, most health management studies based on the UTAUT model tend to place excessive emphasis on motivational variables for technology adoption such as performance expectancy and effort expectancy while paying relatively little attention to the complex interplay between the service delivery environment, users’ personal capabilities, and psychological states. This limitation is particularly pronounced in studies involving older adults, whose decision-making processes regarding the adoption of smart health service systems may be influenced by a broader and more nuanced set of factors. Existing models often fail to adequately capture the synergistic dynamics between older users and the technological or service systems they engage with. Therefore, traditional technology acceptance models may have theoretical limitations in explaining the adoption behavior of SPSS. To gain a more comprehensive understanding of the SPSS adoption mechanisms among the older adults in the context of chronic disease management, it is essential to introduce new theoretical perspectives and constructs. To fill these theoretical gaps.

Firstly, this study introduces Health Consciousness (HC), which aims to measure the level of older adults’ concern for their own health and their proactivity in health-related behaviors. Research has shown that individuals with higher health consciousness are more likely to actively seek health information and adopt digital tools that aid in health management (32). Vervier et al. (33) noted that older adults with high health consciousness exhibit significantly stronger motivation to use mobile health technologies for managing chronic conditions independently. Therefore, health consciousness plays a crucial role in explaining older adults’ intention to use medication management systems.

Secondly, this study incorporates Self-Efficacy (SE) into the extended model. For older adults, the common digital divide and challenges in adapting to new technologies mean that their confidence in operating smart health service systems will directly influence their willingness to adopt such technologies. While some studies Shiferaw et al. (22) have considered self-efficacy, its impact on older adults’ adoption of smart health products, within a comprehensive framework integrating service satisfaction and health consciousness, remains underexplored.

Finally, this study introduces Healthcare Service Satisfaction (MSS). As SPSS is a service delivery platform, the healthcare service experience behind it has a significant influence on older users’ adoption intentions. Existing research has highlighted that factors such as professional competence and the quality of doctor-patient communication significantly affect patient satisfaction, which in turn influences users’ technology adoption behaviors (34). In summary, existing UTAUT-based studies on the adoption of smart health service systems by older adults have limitations in not fully considering key factors such as users’ intrinsic health motivations, operational confidence, and the quality of service experience.

**Figure 1:**
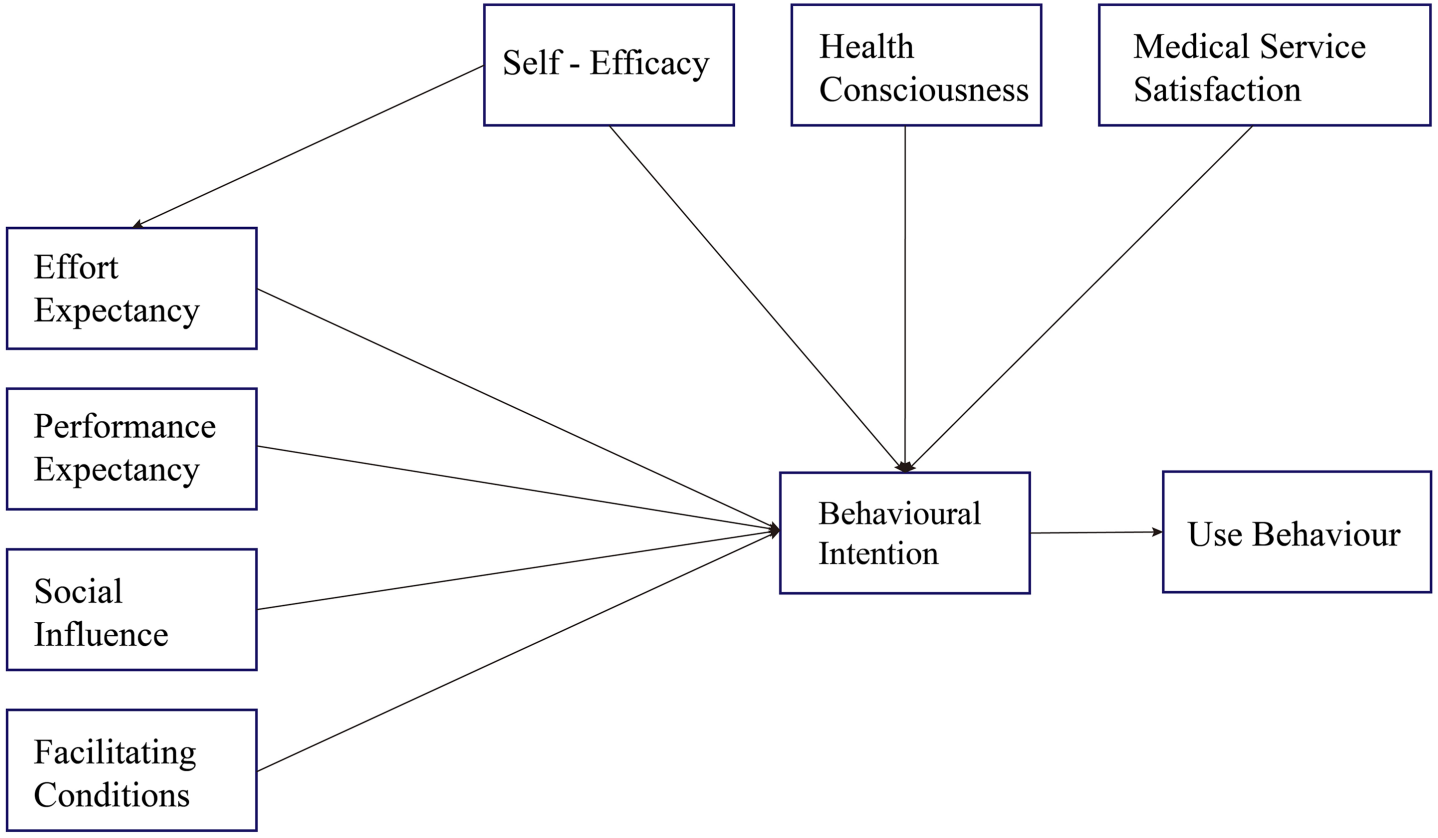
The proposed modified UTAUT model.

This study creatively integrates three variables Health Consciousness (HC), Self-Efficacy (SE), and Medical Service Satisfaction (MSS) into the original UTAUT model. We anticipate that this expanded model will more comprehensively and accurately capture the complex behavioral characteristics influencing the adoption of smart health product service systems by the older adults, thereby providing deeper theoretical insights and practical guidance for understanding and promoting the widespread adoption of digital health services for older adults. The hypotheses are illustrated in Figure 1.

## 3 Hypothesis

Performance expectancy (PE) refers to the extent to which an individual believes that using the system will enhance their task performance (20). In the healthcare domain, users are more likely to show positive intentions to use a system if they believe it will be effective in improving their health management capabilities, such as medication reminders and reducing forgetfulness (35). In medication management systems for older adults, PE has been found to have a significant positive effect on older users’ intention to use smart health devices (9, 36). When older adults believe that the system can assist them in completing medication management more accurately and conveniently, they are more inclined to adopt such systems for daily health support. Therefore, Hypothesis 1 was proposed.

*Hypothesis 1*. PE has a positive impact on the older adults’ behavioral intention to use smart product-service systems.

Effort Expectancy (EE) refers to the perceived ease with which an individual uses a technology system (20). For older users, factors such as ease of use and low learning costs can significantly influence their willingness to use. In the field of health technology, Holden and Karsh (35) noted that the impact of EE is particularly pronounced among healthcare professionals and patient populations, especially when individuals have low familiarity with the technology. Hoque and Sorwar (9) further noted that during the adoption of an mHealth system by older adults, if the system is perceived to be easy to use and does not require too much technical learning, it will greatly increase their willingness to adopt. In addition, Yuan et al. (37) found that EE had a significant effect on increasing older adults’ acceptance of using a medication management system. Hypothesis 2 was proposed.

*Hypothesis 2*. EE has a positive impact on the older adults’ behavioral intention to use smart product-service systems.

Social Influence (SI) refers to the extent to which an individual believes that significant others think they should use a system (20). Research has shown that when older adults perceive that family members or healthcare providers around them positively endorse and recommend medication management technology, they are more likely to accept and try to use the system (23). In addition, Trinh et al. (38) found that technical support and modeling behaviors of family members are guiding for older adults in the adoption process of health technology, and that when promoting medication management systems for older adults, the involvement of family and healthcare providers can significantly enhance users’ motivation to adopt. Hypothesis 3 was proposed.

*Hypothesis 3*. SI has a positive impact on the older adults’ behavioral intention to use smart product-service systems.

Facilitating Conditions (FC) refer to the degree to which an individual believes that the technical and organizational infrastructure exists to support the use of a particular system. According to Venkatesh et al. (20), FC encompass factors such as technical support services, training resources, and users’ technical knowledge. In healthcare systems particularly among older adults populations the support provided by family caregivers and healthcare institutions plays a crucial role in influencing system adoption (39). Hypothesis 4 was proposed.

*Hypothesis 4*. FC has a positive impact on the older adults’ behavioral intention to use smart product-service systems.

Self-efficacy (SE) is a person’s judgments of his or her capacity to engage in behaviors that impact outcomes (40). The degree to which older adults believe they are capable of using SPSS depends on their familiarity with SPSS techniques. Research shows that self-efficacy plays a crucial role in influencing behavioral intentions and effort expectations in various areas of technology. For instance, Rho et al. (41) found that SE had a strong and favorable effect on physicians’ willingness to use telehealth, suggesting that users are more willing to adopt new systems when they feel empowered. Shiferaw et al. (22) noted that by enhancing users’ self-efficacy, healthcare systems can implement telemedicine more smoothly.

*Hypothesis 5*. SE has a positive impact on the older adults’ behavioral intention to use smart product-service systems.

*Hypothesis 6*. SE has a positive impact on older adults’ EE to use smart product-service systems.

Health Consciousness (HC) refers to an individual’s degree of concern about their own health status and their motivation to engage in health-related behaviors (32). Studies have shown that older adults with a higher level of health consciousness are more likely to proactively seek health information and adopt digital tools that assist in health management (42). Especially in contexts where older adult individuals need to independently manage chronic conditions, this self-awareness significantly enhances their motivation to use mobile health systems (43), Cao et al. (44) found that users with high health consciousness tend to hold more positive attitudes toward medication management applications, believing that such systems help improve their quality of life and independence. Hypothesis 7 was proposed.

*Hypothesis 7*. HC has a positive impact on the older adults’ behavioral intention to use smart product-service systems.

Medical Service Satisfaction (MSS) refers to users’ perceptions and evaluations of their experiences within the healthcare system (34). It is also noted that older adults are more likely to embrace and use telehealth technology when they are satisfied with existing medical services. Hypothesis 8 was proposed.

*Hypothesis 8*. MSS has a Positive impact on the older adults behavioral intention to use smart product-service systems.

Behavioral Intention (BI) has been widely recognized as a significant predictor of Use Behavior (UB) across various domains, including both general technology adoption and healthcare-related systems (20, 45). Prior studies have consistently demonstrated that individuals with stronger behavioral intentions are more likely to translate those intentions into actual system use (21). In the context of health information systems, BI has been shown to exert a direct and positive effect on usage behavior, particularly among populations managing chronic conditions or requiring continuous care support (46). Hypothesis 9 was proposed.

*Hypothesis 9*. The behavioral intentions of older people’s SPSS positively influenced the use behavior of SPSS by older people.

## 4 Research methodology

### 4.1 Background on the smart pillbox service system

In this study, the smart pill box service system represents a typical application of SPSS in the healthcare domain. It is specifically designed to support medication management at home for older adults, integrating smart hardware components with continuous digital services to provide a comprehensive medication management solution. The system consists of three core components: (1) Hardware – a smart pill box equipped with sensors to detect medication inventory, visual reminder lights, and audio alarms for scheduled dosing. The device connects to a mobile application via Bluetooth or Wi-Fi, enabling real-time data synchronization; (2) Mobile application – providing personalized medication reminders, adherence tracking, and health status reports; (3) Cloud-based service platform – supporting remote monitoring, data analytics, and bidirectional communication among users, caregivers, and healthcare providers. Through the integration of these hardware and service elements, the system not only assists older adults in accurately following their medication regimens but also delivers timely adherence reports and missed-dose alerts to caregivers.

Unlike conventional pill boxes, the SPSS requires users to engage in continuous interaction with both the physical device and the digital service platform. Users must initially configure their medication schedules, respond to daily reminders, confirm medication intake through the application interface, and regularly review adherence reports. For family members and caregivers, the system provides a dedicated interface with an accessible monitoring dashboard, enabling them to track the medication compliance of older users without intrusive intervention. Healthcare providers can access aggregated adherence data to inform treatment decisions and identify patients who may require additional support.

The dual nature of “product + service” embodies the core value proposition of SPSS, making it an ideal case for examining technology adoption processes that involve both one-time product learning and sustained service engagement. For older adults, this entails not only overcoming initial technological barriers but also developing continuous usage habits and building trust in the service system. The survey conducted in this study was situated within the specific context of this smart pill box service system.

### 4.2 Instrument development

The measuring instrument is one of the most crucial components of the research design, and the validity of the measurement instrument has a direct bearing on the subsequent data analysis. To guarantee the authenticity of the evaluation, I constructed the structured questionnaire based on an analysis of the pertinent literature. The questionnaire items measured the following constructs: the UTAUT construct (PE, EE, SI and FC); and the extended constructs: HC, SE, and MSS. The questionnaire’s scaled items are adapted from Hoque and Sorwar (9), Venkatesh et al. (20), Venkatesh et al. (21). The questionnaire’s content was divided into two sections, A and B. These projects draw on the scales established in relevant studies at home and abroad, and modify them according to the actual situation of the older adults to ensure the reliability and validity of the questionnaires.

Part A covers basic demographic information, including age, gender, education, and mobile phone experience. In addition, a question was set, “Do you know or are you using SPSS,” and the responses were set to “yes” or “no.” If the respondent does not know or has not used SPSS, the respondent will not continue to participate in completing Section B of the questionnaire.

The measurement instruments for testing the model and hypotheses are presented in Section B. measurement items were selected from studies with a similar structure to the current study through a literature review. The survey, consisting of a 5-point Likert scale, indirectly measures the underlying structure through measurement items with scores ranging from 1 (= ‘strongly disagree’) to 5 (= ‘strongly agree’). The relevant measurement items are shown in Table 1.

**Table 1:** Items and contents of measurement constructs.

Construct	Item code	Corresponding item
Performance Expectation (PE) (20, 69)	PE1	I find the Smart Pill Box Service System (SPSS) useful in my daily life.
	PE2	Using the Smart Pill Box Service System (SPSS) can help me complete tasks faster.
	PE3	Using the Smart Pill Box Service System (SPSS) can improve my medication management efficiency.
	PE4	Using the Smart Pill Box Service System (SPSS) can improve my medication management.
Effort Expectancy (EE) (21)	EE1	Learning how to use the Smart Pill Box Service System (SPSS) was easy for me.
	EE2	My interaction with the Smart Pill Box Service System (SPSS) is clear and easy to understand.
	EE3	I found the Smart Pill Box Service System (SPSS) to be easy to use.
	EE4	I can easily and skillfully use the Smart Pill Box Service System (SPSS).
Social Influence (SI) (21, 69)	SI1	People who are important to me think I should use the Smart Pill Box Service System (SPSS).
	SI2	People who influence my behavior think I should use the Smart Pill Box Service System (SPSS).
	SI3	I value opinions more than I use the Smart Pill Box Service System (SPSS).
	SI4	Overall, my healthcare facility has been supporting and encouraging the use of the Smart Pill Box Service System (SPSS).
Facilitating Conditions (FC) (20)	FC1	I have the resources I need to use the Smart Pill Box Service System (SPSS).
	FC2	I have the knowledge I need to use the Smart Pill Box Service System (SPSS).
	FC3	The Smart Pill Box Service System (SPSS) is compatible with other technologies I use.
	FC4	Experts and medical staff can help me solve problems encountered with the Smart Pill Box Service System (SPSS).
	FC5	I have knowledge resources (manuals, documentation) to support me in using the Smart Pill Box Service System (SPSS).
Health Consciousness (HC) (70, 71)	HC1	I am very conscious about maintaining my health in daily life.
	HC2	I pay close attention to information about health and wellness.
	HC3	I actively try to avoid behaviors that could harm my health.
	HC4	I regularly take actions to improve my health, such as taking prescribed medications.
	HC5	I am concerned about the impact of my lifestyle choices on my long-term health.
Self-Efficacy (SE) (72)	SE1	I have the confidence to use the Smart Pill Box Service System (SPSS) even if no one tells me how to do it.
	SE2	I am confident in using the Smart Pill Box Service System (SPSS) even though I have never used this system before.
	SE3	I am confident in using the Smart Pill Box Service System (SPSS) even though I only have a reference in the software manual.
	SE4	If I have enough time, I can complete the tasks provided by the system.
Behavioral Intention (BI) (20)	BI1	If I have access to the Smart Pill Box Service System (SPSS), I intend to use this service in the future.
	BI2	If I have the opportunity to access the Smart Pill Box Service System (SPSS), I will consistently try to use it in my daily life.
	BI3	I plan to use the Smart Pill Box Service System (SPSS) in the future.
Usage Behavior (UB) (20)	UB1	Using the Smart Pill Box Service System (SPSS) is a pleasant experience.
	UB2	I currently use the Smart Pill Box Service System (SPSS).
	UB3	I spend a lot of time on the Smart Pill Box Service System (SPSS).
	UB4	I have been using the Smart Pill Box Service System (SPSS).
Medical Service Satisfaction (MSS) (73–74)	MSS1	I am satisfied with the overall service provided by the Smart Pill Box Service System (SPSS).
	MSS2	The Smart Pill Box Service System (SPSS) helps me manage my medication effectively.
	MSS3	The instructions and information provided by the system are clear and easy to understand.
	MSS4	The system is reliable and functions consistently without errors.
	MSS5	Using the Smart Pill Box Service System (SPSS) saves me time and effort in managing my medication.
	MSS6	I feel confident and safe using the Smart Pill Box Service System (SPSS) for my daily health needs.

### 4.3 Data collection

In this study, the data collection process consisted of two main phases: the preliminary survey and the formal survey. During the preliminary survey, two peer experts were invited to evaluate the initial version of the questionnaire, focusing on its content structure and clarity. A pretest was conducted with 10 older adult individuals to ensure the sample included participants aged 60 and above. The pretest aimed to assess the comprehensibility of the questionnaire, and based on feedback from older adults participants, revisions were made to refine the questionnaire, resulting in the final version. During the translation process, the research team adopted a two-way translation method, translating the original English questionnaire into Chinese, and another person familiar with English translated the Chinese version back into English, and then compared and revised the English version of the translation with the original English version. Check for any discrepancies in meaning or content. We adopted a forward–backward translation with team reconciliation to ensure semantic equivalence. To address potential dialectal variation, the instrument was administered in standard written Chinese; a cognitive pretest with older adults confirmed item clarity and revealed no dialect-related comprehension issues. The formal survey phase employed convenience sampling to select older adult individuals as potential users of the Smart Product Service System (SPSS) for medication management. Participants were recruited from community groups within medical institutions in China, and an online survey was distributed to allow older adult individuals or their children to complete the questionnaire based on their actual circumstances, ensuring linguistic and cultural appropriateness. Inclusion criteria included individuals aged 60 and above, without severe cognitive impairments, and who were willing to participate in the study.

Data collection was conducted from April 18, 2022, to May 29, 2022. At the start of the online survey, participants were provided with an information statement outlining the study’s purpose, the voluntary nature of participation, the right to withdraw at any time, and data confidentiality, while ensuring that the survey did not involve medical interventions or psychological risks. No personal identifying information was collected. Completing the survey was considered an indication of informed consent. In March 2022, the ethics committee of the School of Arts at Beijing Union University reviewed and approved this consent procedure.

A total of 417 participants were recruited, and the survey lasted for 1 month. To ensure the validity of the questionnaire, the data were screened for anomalies. This involved checking for abnormal patterns in the responses from the 417 samples. The criteria for excluding invalid responses included: first, identifying questionnaires with anomalous patterns in consecutive answers, such as when more than 20 consecutive answers were identical, in which case the data were considered invalid. Second, the average time taken by respondents to complete the questionnaire was observed. If the time was significantly shorter or longer than the average, the responses were considered invalid. Given the length of the questionnaire, some participants may have experienced fatigue or provided irrelevant answers. Therefore, any questionnaires that met these criteria were excluded. Based on these standards, 25 samples with anomalous responses were excluded, resulting in a valid sample size of *n* = 392 for data analysis. In addition, to control for non-response bias, this study adopted multiple reminders and supplementary strategies during data collection to maximize response rates.

## 5 Analysis methods

In this study, Structural Equation Modeling (SEM) was employed to validate the structured data. A widely used method within SEM is Partial Least Squares (PLS) regression, chosen for several key reasons.

Firstly, PLS is particularly beneficial in the early stages of theoretical model development, especially when the measurement model and its constructs are not yet fully established (47). Secondly, PLS allows for the measurement of both reliability and validity in structural models, providing insights into the relationships between various factors in the measurement model. This makes PLS particularly suitable for testing complex theoretical models, such as the expanded UTAUT framework.

Therefore, in this study, PLS regression was utilized to verify and test the hypothesized relationships within the expanded UTAUT model, enabling a robust analysis of the factors influencing older adult users’ adoption of smart product-service systems for medication management.

## 6 Results

### 6.1 Demographic information

Table 2 presents the demographic data of the older adults participants in the survey. From the table, it can be seen that of the 392 older adults participants in the survey, 199 of the sample were male, accounting for 50.77% of the total number of respondents, and 193 of the sample were female, accounting for 49.23% of the total number of respondents.

**Table 2:** Demographic and social characteristics of participants.

Items	Categories	N	Percent (%)	Cumulative percent (%)
Gender	Male	199	50.77	50.77
	Female	193	49.23	100.00
Age	60–64 years old	98	25.00	25.00
	65–69 years old	103	26.28	51.28
	70–75 years old	104	26.53	77.81
	76 over years old	87	22.19	100.00
Education level	High school and below	186	47.44	47.44
	Undergraduate	104	26.53	73.97
	Postgraduate	91	23.21	97.18
	PhD and above	11	2.82	100
Experience with mobile phones	1 year	90	22.96	22.96
	1–2 years	101	25.77	48.72
	3–4 years	110	28.06	76.79
	More than 5 years	91	23.21	100.00
Total		392	100.0	100.0

The results of the age distribution range were: 98 people were between 60 and 64 years old, representing 25.0% of the total number of participants. One hundred three people were between 65 and 69 years old, representing 26.28% of the total number of participants. One hundred four people were between 70 and 75 years old, representing 26.53% of the total number of participants. One hundred three people were between 65–69 years old, accounting for 26.28% of the total number of participants. One hundred four people were between 70 and 75 years old, accounting for 26.53% of the total number of participants. Eighty-seven people were over 76 years old, accounting for 22.19% of the total number of participants.

The majority of the seniors surveyed had an education level of high school or below, accounting for 47.44% of the total number of respondents. This was followed by 104 seniors with a bachelor’s degree, accounting for 26.53% of the total number of respondents. Ninety-one seniors had a postgraduate degree, accounting for 23.21% of the total number of respondents. Eleven seniors had a doctorate degree, accounting for 2.82% of the total number of respondents. Based on the results of the data, it can be seen that there is a wide variation in the level of education received by the older adults in the sample data.

It can be seen that 90 of the seniors who participated in the survey have been using mobile phones for 1 year, accounting for 22.96% of the total, and 101 of the seniors who participated in the survey have been using mobile phones for 1–2 years, accounting for 25.77% of the total, 110 of the seniors who participated in the survey have been using mobile phones for 3–4 years, accounting for 28.06% of the total, and 91 of the seniors who participated in the survey have been using mobile phones More than 5 years, accounting for 23.21% of the total number of participants.

### 6.2 Measurement models

To assess the presence of common method variance (CMV) and multicollinearity in the study data, the Full Collinearity VIF approach proposed by Kock (48) was employed. This method treats all latent variables as dependent variables in a full collinearity test and calculates the variance inflation factor (VIF) values for both the outer measurement model (Outer VIF) and the inner structural paths (Inner VIF). According to Kock (48), VIF values below 3.3 indicate the absence of serious common method bias and acceptable levels of multicollinearity.

The results show that all Outer VIF values for the measurement indicators in this study range from 1.747 to 2.791, well below the threshold of 3.3 (see Table 3), suggesting that neither substantial common method bias nor problematic multicollinearity exists. Similarly, the Inner VIF values for the structural model paths range from 1.000 to 1.349, also below 3.3, further supporting the robustness of the model.

**Table 3:** Full collinearity VIF results.

Construct and measurement indicators	Outer VIF
Behavioral Intention (BI)	
BI1	1.959
BI2	1.874
BI3	2.188
Effort Expectancy (EE)	
EE1	1.860
EE2	2.568
EE3	2.223
EE4	1.934
Facilitating Conditions (FC)	
FC1	1.962
FC2	2.181
FC3	2.791
FC4	1.923
FC5	2.040
Health Consciousness (HC)	
HC1	2.738
HC2	1.886
HC3	1.947
HC4	1.860
HC5	2.041
Mobile Service Satisfaction (MSS)	
MSS1	1.961
MSS2	2.710
MSS3	1.749
MSS4	1.875
MSS5	2.164
MSS6	1.944
Performance Expectancy (PE)	
PE1	1.905
PE2	1.929
PE3	2.136
PE4	1.940
Self-Efficacy (SE)	
SE1	2.058
SE2	2.109
SE3	2.631
SE4	1.925
Social Influence (SI)	
SI1	1.828
SI2	1.793
SI3	1.747
SI4	2.321
Use Behavior (UB)	
UB1	1.920
UB2	2.437
UB3	1.972
UB4	1.887
Path relationship	Inner VIF
BI → UB	1.000
EE → BI	1.321
FC → BI	1.157
HC → BI	1.316
MSS → BI	1.349
PE → BI	1.304
SE → BI	1.291
SE → EE	1.000
SI → BI	1.263

All VIF values are below 3.3 (48), indicating that there is no serious common method bias (CMV) and multicollinearity problems in the data.

Validating the measurement model’s dependability requires looking at the convergent validity, discriminant validity, and internal consistency of the construct (49). Cronbach’s alpha, composite reliability (CR), and extracted mean–variance (AVE) of the analysis results are used as metrics to examine convergent validity, discriminant validity, and internal consistency, respectively (75). Below are the values of their test metrics.

The examination of internal reliability utilized Cronbach’s alpha, and it was decided that a value of Cronbach’s alpha over 0.70 for the measurement model was an acceptable sign of internal consistency (51). According to Table 4, the computed Cronbach’s alpha values range between 0.838 and 0.889. This is above the acceptable threshold, indicating that the prerequisites for internal reliability have been met.

**Table 4:** The measurement model.

Construct	Items	Loadings	AVE	CR	Cronbach’s alpha
Performance expectancy	PE1	0.761	0.598	0.856	0.855
	PE2	0.769			
	PE3	0.790			
	PE4	0.775			
Effort expectancy	EE1	0.743	0.627	0.870	0.868
	EE2	0.841			
	EE3	0.810			
	EE4	0.769			
Social influence	SI1	0.750	0.627	0.847	0.844
	SI2	0.738			
	SI3	0.733			
	SI4	0.824			
Facilitating conditions	FC1	0.751	0.612	0.887	0.885
	FC2	0.789			
	FC3	0.860			
	FC4	0.741			
	FC5	0.763			
Health consciousness	HC1	0.852	0.594	0.879	0.878
	HC2	0.741			
	HC3	0.752			
	HC4	0.733			
	HC5	0.768			
Self-efficacy	SE1	0.783	0.634	0.873	0.871
	SE2	0.792			
	SE3	0.845			
	SE4	0.762			
Behavioral intention	BI1	0.830	0.635	0.839	0.838
	BI2	0.759			
	BI3	0.800			
Medical service satisfaction	MSS1	0.745	0.576	0.890	0.889
	MSS2	0.839			
	MSS3	0.705			
	MSS4	0.723			
	MSS5	0.791			
	MSS6	0.742			
Use Behavior	UB1	0.768	0.609	0.861	0.860
	UB2	0.823			
	UB3	0.758			
	UB4	0.769			

AVE, average variance extracted; CR, composite reliability.

The degree of convergent validity was determined by multiplying the extracted variance (AVE), and the level of meeting convergent validity was determined by requiring each item to have an AVE of at least 0.50 (52). When the composite reliability (CR) score for each factor is larger than 0.70 and the extracted average variance (AVE) value is larger than 0.50, convergent validity is generally regarded as good. According to Table 4, the AVE values ranged between 0.576 and 0.634. The composite reliability (CR) values ranged from 0.839 to 0.890, with values for both indicators exceeding the recommended range. Consequently, it may be argued that the current survey satisfies the criteria for convergent validity.

In contrast, the square root of the AVE and the cross-load matrix were utilized to determine the test’s discriminant validity. For a concept to have sufficient discriminant validity, the square root of its AVE must’ve been larger than its correlations with other constructs. This is the metric used to evaluate discriminant validity.

Table 5 displays the test results in accordance with the discriminant validity that was determined. If the constructed correlation between the variables is greater than the square root of the value of the AVE that is shown on the diagonal between the variables that were tested, then discriminant validity is not present; on the other hand, discriminant validity is demonstrated if the situation is the opposite. For instance, a correlation of 0.156 between EE and SI will never surpass the square root of the AVE values of EE and SI, which is 0.792 (i.e., 0.762). Since the discriminant validity of the inferred components was effectively fulfilled, it was proved that the data for this model structure had discriminant validity.

**Table 5:** Correlation matrix and square root of the AVE.

	PE	EE	SI	FC	HC	SE	BI	MSS	UB
PE	**0.774**								
EE	0.272***	**0.792**							
SI	0.346***	0.156**	**0.762**						
FC	0.203***	0.229***	0.226***	**0.782**					
HC	0.408***	0.317***	0.288***	0.244***	**0.770**				
SE	0.304***	0.269***	0.363***	0.197***	0.300***	**0.796**			
BI	0.471***	0.398***	0.411***	0.227***	0.429***	0.488***	**0.797**		
MSS	0.320***	0.434***	0.261***	0.240***	0.180**	0.333***	0.180**	**0.759**	
UB	0.277***	0.393***	0.338***	0.267***	0.267***	0.375***	0.267***	0.441***	**0.780**

Bold diagonal values represent the square root of the Average Variance Extracted (AVE). ** *p* < 0.01; *** *p* < 0.001 (two-tailed).

Table 6 metrics are used to evaluate the measurement model’s goodness-of-fit. The metrics presented in Table 6 is used to evaluate the measurement model’s overall fit. When all of these measurements fall within the range of values shown in the table, a measurement model is deemed to be well-fitting (53).

**Table 6:** Model evaluation metrics and recommended values.

Index	*χ*^2^/df	CMIN/DF	NFI	IFI	TLI	CFI	AGFI	RMSEA
Model value	*χ*^2^ = 1019.467 df = 678.00	1.504	0.882	0.957	0.953	0.957	0.883	0.036
Recommended value		<3 good fit	>0.8	>0.9	>0.8	>0.9	>0.8	<0.05 good fit <0.10 reasonable fit

We can see all of the model fit indices and the numerical recommendation criteria for this study in Table 6. A value of 1019.467, 678.00, and 1.504 for the chi-squared degree of freedom were obtained from the total measured model fit index test. It was discovered that a chi-squared/DF value of less than 3.0 fell within the suggested range of values (54). Because it is possible to base it on the comparison of the numerical results provided in Table 6 with the values of the indicated range, it is possible to draw the conclusion that the measurement model is appropriate for further inquiry based on the findings of this investigation. The model has achieved an acceptable degree of fit and is now ready for the next stage of examination.

To ensure the robustness of the research findings, the structural model was further subjected to robustness testing by comparing a reduced model with the full structural model. Following the recommendations of Hair et al. (55) and Shmueli et al. (56). The reduced model was derived directly from the original UTAUT framework, retaining only the baseline constructs and hypothesized relationships, Performance Expectancy (PE), Effort Expectancy (EE), Social Influence (SI), and Facilitating Conditions (FC), as predictors of Behavioral Intention (BI) and Use Behavior (UB). It excluded all three extended constructs introduced in this study, namely Health Consciousness (HC), Medical Service Satisfaction (MSS), and Self-Efficacy (SE), along with their respective hypothesized paths (HC → BI, MSS → BI, SE → BI, and SE → EE).

In contrast, the full model incorporates these additional constructs and paths to capture service-system contextual factors that are specific to the Smart Pillbox Service System. This comparative design enables a direct evaluation of whether including these context-specific variables materially enhances explanatory and predictive performance.

The comparison results (Table 7) indicate that the full model provides substantially stronger explanatory power for Behavioral Intention (BI), with *R*^2^ increasing from 0.294 in the reduced model to 0.379 in the full model, while also accounting for an additional 7.3% of the variance in Effort Expectancy (EE). Moreover, predictive relevance (*Q*^2^ predict) results reveal that the full model consistently outperforms the reduced model in terms of BI indicators, with lower Root Mean Square Error (RMSE) values, thereby enhancing predictive accuracy. In addition, all newly added paths (HC → BI, MSS → BI, SE → BI, and SE → EE) were statistically significant (*p* < 0.01), highlighting their critical role in explaining technology adoption within a service-context framework. Taken together, these findings demonstrate that incorporating the service-related constructs improves model fit, explanatory power, and predictive validity, supporting the retention of these paths in the final model (47).

**Table 7:** Comparison between full model and reduced model.

Indicator	Full model	Reduced model	Change/Interpretation
*R*^2^ (BI)	0.379	0.294	Increased explanatory power
Adjusted *R*^2^ (BI)	0.368	0.286	Increased
*R*^2^ (UB)	0.160	0.159	Essentially unchanged
Adjusted *R*^2^ (UB)	0.157	0.157	No change
*R*^2^ (EE)	0.073	—	Newly explained by additional path
*Q*^2^ predict (BI, average)	0.250	0.204	Improved predictive relevance
*Q*^2^ predict (UB, average)	0.114	0.099	Improved
Number of significant paths	7/9	4/5	More significant relationships detected
Highest path coefficient	0.399 (BI → UB)	0.399 (BI → UB)	Identical
Prediction error (BI1 RMSE)	0.991	1.036	Reduced error
Prediction error (UB1 RMSE)	1.062	1.078	Reduced error

The reduced model follows the baseline UTAUT framework without the additional service-related constructs, whereas the full model incorporates HC, MSS, and SE along with their direct and mediating paths.

### 6.3 Structural equation modeling

This study use structural equation modeling to investigate the link between the variables’ impacts (Table 8). The indicators it assessed was based on the t-statistic and the path coefficient (*β*) (57, 58).

**Table 8:** Structural model.

Hypothesis	Path	*β*	*t*-statistics	*P*	Comments
H1	PE- > BI	0.189	3.244	0.001	Supported
H2	EE- > BI	0.156	3.127	0.002	Supported
H3	SI- > BI	0.161	2.89	0.004	Supported
H4	FC- > BI	0.009	0.189	0.85	Not supported
H5	SE- > BI	0.247	4.185	***	Supported
H6	SE- > EE	0.292	4.993	***	Supported
H7	HC- > BI	0.169	3.09	0.002	Supported
H8	MSS- > BI	0.175	3.282	0.001	Supported
H9	BI- > UB	0.486	8.065	***	Supported

Significant at *p* < 0.05. *** *p* < 0.001 (two-tailed). All hypotheses were tested at a significance level of *p* < 0.05.

The outcomes of the path analysis, among other things, shed light on the influence of variables on the degree of structure or effect size, as indicated by a correlation estimate β above 0.30, indicating a strong influence. We can observe that the analysis yields a significant association between the use behavior (UB) and behavioral intention (BI) components with *β* = 0.486, indicating that the relationship for H9 has a substantial effect size.

Using the magnitude of the t-statistic and the value of *P*, the significance of the hypothesis may be determined. 1.96 (level of significance = 5%) was the value of the *t*-statistic., 2.85 (level of significance = 1%) and 3.29 (level of significance = 0.1%) (47).

Therefore, a *t*-value of 1.96 or higher, *p* > 0.05, would suggest that the relationship between the influence of components is significant and would support the study’s hypothesis, whereas a *t*-value of 1.96 or less, *p* > 0.05, would show that there is no significant effect and would not (57, 58).

The path coefficients’ estimated values and test statistics are displayed in Table 8. We discovered that eight out of the nine route links gained significance for the hypothesis estimating the path associations between each pair of study components using PLS regression. Below is a summary of them.

Based on the data, the following hypothesis (H1, H2, H3, H5, H6, H7, H8, and H9) were supported. The pathways PE-BI (*t* = 3.244, *B* = 0.189, *p* < 0.05), EE-BI (*t* = 3.127, *B* = 0.156, *p* < 0.05), SI-BI (*t* = 2.89, *B* = 0.161, *p* < 0.05), SE-BI (*t* = 4.185, *B* = 0.247, *p* < 0.05), SE-EE (*t* = 4.993, *B* = 0.292, *p* < 0.05), HC-BI (*t* = 3.09, *B* = 0.169, *p* < 0.05), MSS-BI (*t* = 3.282, *B* = 0.175, *p* < 0.05), BI-UB (*t* = 8.065, *B* = 0.486, *p* < 0.05).

The SE, EE, PE, SI, and MSS had a favorable relationship with users’ behavioral intention to use SPSS for medication management in the older adults. In addition, HC has a positive effect on users’ behavioral intent to use SPSS. However, the path from FC to BI (*t* = 0.189, = 0.009, *p* > 0.05) exhibited unimpressive results; hence H4 was not supported by this investigation. Therefore, it can be demonstrated that FC has no influence on the behavioral intention of the older adults to use SPSS for medication management.

The results of the indirect and total effects are presented in Table 9. Bootstrapping analysis (5,000 resamples) indicates that the indirect effect of self-efficacy (SE) on behavioral intention (BI) through effort expectancy (EE) is significant (*β* = 0.035, *t* = 2.418, *p* < 0.05), suggesting that self-efficacy can influence behavioral intention indirectly via effort expectancy. In addition, the total effect of SE on BI is also significant (*β* = 0.258, *t* = 5.443, *p* < 0.001), indicating that self-efficacy not only exerts a direct impact on behavioral intention but also affects it indirectly through the mediating pathway.

**Table 9:** Indirect effects and total effects (bootstrapping, 5,000 resamples).

Path	Original sample (β)	Sample mean	Std. dev.	*t*-value	*p*-value	Significance
Indirect effects						
SE → EE → BI	0.035	0.035	0.014	2.418	0.016	*
Total effects						
SE → BI	0.258	0.257	0.047	5.443	0.000	***

* *p* < 0.05; *** *p* < 0.001 (two-tailed). Significant at *p* < 0.05.

## 7 Discussion

This study is theoretically grounded in the UTAUT model and examines the variables influencing older adults’ BI to use SPSS for medication management. An extended model was developed based on a comprehensive literature review. The proposed extended UTAUT model consists of both core constructs EE, PE, FC, and SI and additional constructs HC, SE, and MSS.

According to the research findings, all proposed factors except FC showed significant associations with older adults’ behavioral intention to use SPSS. These results indicate that older adults’ intention to adopt SPSS for medication management is influenced by multiple factors, which will be discussed in detail in the following sections.

First, by testing hypotheses H1–H4, we validated the impact of core driving factors in the UTAUT model on behavioral intention to use technology. The study found that PE, EE, and SI have a significant positive effect on older adults’ behavioral intention to use SPSS. This result is consistent with the findings of several previous studies based on the UTAUT model (50, 76).

Venkatesh et al. (20) identified in the original UTAUT model that PE and EE are key variables in predicting technology adoption intention. In this study, older adults’ PE and EE regarding the SPSS are reflected in their trust that the system will enhance health management efficiency and reduce the likelihood of forgetting to take medication. This finding is consistent with the results of Cimperman et al. (59), who found that older adult individuals are more likely to adopt telemedicine systems if they believe that such systems will improve their health management efficiency. This study also supports the findings of Duarte and Pinho (60) in their research on consumer adoption of mobile health technologies, which concluded that simplicity, user-friendliness, and intuitive information presentation are key factors driving the adoption of smart health devices and increasing users’ behavioral intention. Additionally, the significant role of SI aligns with the phenomenon of “strong family dependence” observed in East Asian cultures. As noted by Wei et al. (8), in family-centered societies, the opinions of family members and caregivers have a strong influence on the technology adoption behavior of older adult individuals. In this study, the significant impact of SI further confirms that, in China, older adults’ adoption of health-related smart technologies is often influenced by recommendations from family members and community healthcare professionals. This trend is also reflected in the study by Lan et al. (61), which examined the adoption of mobile health (mHealth) services among patients with chronic diseases in the context of aging and the high prevalence of chronic conditions.

This study found that although FC is considered a key factor influencing behavioral intention in the UTAUT model (20), FC did not have a significant impact on the intention to use the SPSS among the older adults participants in this study. This result is consistent with some previous studies. Shiferaw et al. (22) indicated that FC can enhance healthcare providers’ positive attitudes toward telemedicine; however, FC did not directly and significantly affect users’ behavioral intention when it came to actual adoption of telemedicine services. Hoque and Sorwar (9) further proposed that FC may be influenced by other factors, such as PE or EE, and when these factors are present in the research model simultaneously, the impact of FC on behavioral intention may become insignificant. A possible explanation for this discrepancy is that, in the cultural and social context of this study, older adult individuals tend to rely more on the technological assistance of family members rather than viewing institutional or infrastructural support as a decisive factor. Hunsaker et al. (62) emphasized that older adults who are less familiar with digital technology may have specific concerns about the technology’s usability. In family-centered social structures, older adult individuals are more likely to accept technological advice and assistance from relatives rather than support provided by institutions or platforms. A systematic review by Yap et al. (63) further pointed out that many older adult individuals, being unfamiliar with digital technology, focus on usability issues during the initial usage phase and are more reliant on their own experiences or guidance from those around them to overcome technological barriers. Therefore, although the UTAUT model considers FC as a key influencing factor, in specific populations and cultural contexts, the role of FC may be mediated or replaced by variables such as family support and social influence, which could diminish its significance in influencing older adults’ behavioral intention to use SPSS. In the extended constructs, the results confirmed that the three extended factors SE, MSS, and HC all showed significant effects. Among them, SE was found to be an important predictor of behavioral intention, which is consistent with previous research. Mensah et al. (64) pointed out that self-efficacy plays a key role in explaining the adoption of e-health technologies by older adults. In the Chinese context, older adult individuals have long been influenced by the stereotype that “technology is for young people,” leading to a certain degree of apprehension when confronted with emerging smart products. Therefore, enhancing SE can help break through these traditional conceptual limitations and increase their initiative and willingness to engage with new technologies. MSS plays a critical role as a predictor and explanatory factor for older adult users’ adoption of SPSS. The results align with previous findings in the context of health technology adoption, where increasing older adult individuals’ satisfaction with healthcare services and the quality of care they receive can enhance the use of telemedicine (34). In the case of chronic disease management, when users are satisfied with their overall experience with the system, their behavioral intention to use the system is more likely to increase.

This study found that HC has a significant positive impact on older adults’ behavioral intention to use SPSS. This result is consistent with previous research (65, 66). older adult individuals with higher health consciousness are more inclined to actively seek health-related information and adopt technologies that aid in disease prevention, medication adherence, and health monitoring. This suggests that a strong sense of health consciousness can motivate them to explore digital tools that support independent health management. The finding that behavioral intention significantly influences usage behavior is consistent with previous UTAUT-based research across different fields. This further confirms that the greater the individual’s intention to use a technology, the more likely they are to actually use the system. In the context of older adult individuals using SPSS for medication management, behavioral intention becomes a key factor in predicting actual usage behavior. Previous studies have indicated that when older adult users perceive the system as helpful for health management, easy to operate, and tailored to their needs, their intention to use the system is more likely to translate into continued usage behavior (9, 59).

Finally, this study highlights the mediating role of EE in the relationship between SE and BI to adopt SPSS. SE shapes adoption intention through perceptions of ease of use, which aligns with the cognitive appraisal mechanism proposed in social cognitive theory (67). In the context of chronic disease management, a similar mediation pattern has been validated: when Chinese patients use mobile health services, self-efficacy influences adoption intention effectively only through effort expectancy (68). Moreover, this study identified a partial rather than full mediation effect, indicating that self-efficacy still exerts a significant direct impact on behavioral intention. This may reflect the influence of specific cultural values among older adults in China, whereby self-efficacy affects technology adoption decisions not only indirectly via effort expectancy but also directly.

## 8 Contribution

This study addresses the problem of low medication adherence for chronic diseases among Chinese older adults, and focuses on the intention of Chinese older adults to use the SPSS to manage their medication behaviors, adopting the extended UTAUT model with the introduction of external variables such as SE, HC, and MSS, which has the following theoretical and practical contributions:

First, by validating the pathways through which SE, HC, and MSS influence the behavioral intention of older adults, this study extends the original UTAUT model, enhancing its explanatory power in the context of older adults health technology adoption. Based on a sample of older adult individuals from China, this research compares the application of the UTAUT model to previous studies predominantly conducted in Western contexts, providing empirical support for the model’s applicability and limitations in non-Western cultural settings.

Second, this study thoroughly examines the influence of China’s unique cultural context on older adults’ adoption of SPSS and provides culturally specific insights. The findings offer data-driven quantitative research and pathways for future SPSS development, which can guide designers and developers in optimizing their products. These results are expected to significantly improve older adult users’ acceptance, enhance their digital quality of life, and, through user-friendly design, increase satisfaction. Ultimately, this will help drive technology adoption, benefiting broader public health.

## 9 Limitation

Firstly, the data collection for this study was conducted online and was limited to older adults without cognitive impairments who used mobile devices and smartphones. From the perspective of data completeness, future research should consider including older adults who face difficulties in using information technology, as they play a crucial role in user experience and the implementation of accessible product design. Therefore, future studies could expand the sample to include these groups to ensure the comprehensiveness of the research. Additionally, future research could adopt longitudinal or experimental designs, such as implementing SPSS interventions in different older adults communities, to provide stronger evidence for causal inferences.

Second, this study did not use SPSS or structural equation modeling tools to further examine the moderating effects of four control variables (e.g., gender, age, education level, and health status) between exogenous variables and behavioral intention. Therefore, it is unclear whether these variables significantly moderate the technology adoption path across different groups. Future research could adopt a moderated mediation model to explore the role of these control variables within the extended UTAUT model, thus gaining a more accurate understanding of the heterogeneity in older adults’ technology adoption behaviors.

Third, this study only employed quantitative research methods to explore the factors influencing the use of SPSS for medication management among older adult individuals. However, it is challenging to explain the issues that arise when older adult users actually use the technology. Therefore, future research should adopt a mixed-methods approach to further investigate older adult individuals’ perspectives and gain deeper insights into the reasons that affect their adoption of SPSS for medication management.

## Data Availability

The datasets presented in this study can be found in online repositories. The names of the repository/repositories and accession number(s) can be found at: https://figshare.com/s/ff4fe198c78ee98c73f8.
